# Targeting NPM1 in irradiated cells inhibits NPM1 binding to RAD51, RAD51 foci formation and radiosensitizes NSCLC

**DOI:** 10.1016/j.canlet.2020.12.023

**Published:** 2020-12-21

**Authors:** Geri Traver, Konjeti R. Sekhar, Peter A. Crooks, Diane S. Keeney, Michael L. Freeman

**Affiliations:** a Department of Radiation Oncology, Vanderbilt University Medical Center, Nashville, TN, 37232, USA; b Department of Pharmaceutical Sciences, College of Pharmacy, University of Arkansas for Medical Sciences, Little Rock, AR72205, USA; c Cumberland Emerging Technologies, Inc., 2525 West End Ave, Suite 950, Nashville, TN, 37203-1608, USA

**Keywords:** Nucleophosmin1, YTR107, Radiation sensitization, RAD51, NSCLC

## Abstract

The ability of chemo-radiation therapy to control locally advanced stage III non-small cell lung cancer (NSCLC) is poor. While addition of consolidation immunotherapy has improved outcomes in subsets of patients there is still an urgent need for new therapeutic targets. Emerging research indicates that nucleophosmin1 (NPM1) is over-expressed in NSCLC, promotes tumor growth and that over-expression correlates with a lower survival probability. NPM1 is critical for APE1 base excision activity and for RAD51-mediated repair of DNA double strand breaks (DSBs). YTR107 is a small molecule radiation sensitizer that has been shown to bind to NPM1, suppressing pentamer formation. Here we show that in irradiated cells YTR107 inhibits SUMOylated NPM1 from associating with RAD51, RAD51 foci formation and repair of DSBs. YTR107 acts synergistically with the PARP1/2 inhibitor ABT 888 to increase replication stress and radiation-induced cell lethality. YTR107 was found to radiosensitize tumor initiating cells. Congruent with this knowledge, adding YTR107 to a fractionated irradiation regimen diminished NSCLC xenograft growth and increased overall survival. These data support the hypothesis that YTR107 represents a therapeutic target for control of NSCLC.

## 1. Introduction

Concurrent chemoradiation (CRT) followed by immunotherapy is standard of care for most patients with locally advanced stage II-III non-small cell lung cancer (NSCLC) [1,2]. CRT by itself poorly controls tumor growth and subsequent metastases [3]: median survival is approximately 24 months; 5-year survival about 17% [4]. For patients whose tumors did not progress after CRT, adding consolidation immunotherapy yielded an overall survival of approximately 50% at 36 months [5]. Although encouraging, outcomes for locally advanced stage II-III NSCLC illustrate the unmet need to significantly improve therapeutic response, especially for patients whose tumors progress during CRT (approximately 30% of tumors progress within the radiation field [6]) and for those whose overall survival following CRT plus immunotherapy is less than 50%.

Cancer stem cells, or perhaps a more accurate term is tumor initiating cells (TICs), are considered a subpopulation that contributes to tumor progression during therapy or tumor recurrence after therapy [7]. Emerging evidence indicates TICs mount overly robust DNA Damage Responses (defined in ref 7) that confer intrinsic radioresistance to these cells. Increased intrinsic radioresistance to DNA double strand breaks can increase TIC survival probability [7]. DDR pathways include base excision repair, nucleotide excision repair, translesion synthesis, mismatch repair, non-homologous end joining, alternative non-homologous end joining, single strand annealing, and homologous recombination (HR). These highly conserved pathways are considered actionable targets for tumor initiating cells [8] and cancer therapy in general [9].

Nucleophosmin1 (NPM1) is a DNA, RNA and protein chaperone involved in cell cycle regulation, centrosome duplication, ribosome biogenesis, and the DDR (reviewed in Ref. [10]). NPM1 is over expressed in NSCLC [11,12] and its over expression correlates with lower overall survival in NSCLC, as well as in renal, liver, and head and neck cancers [11]. Preclinical analysis supports the concept that NPM1 is a therapeutic vulnerability for KRAS mutated NSCLC [11].

NPM1 has crucial roles in base excision, translesion synthesis and the HR DDR pathways [13–15]. Phosphorylation of NPM1 at Thr199 by CDK2/cyclin E [16] and SUMOylation at K263 by Arf [17] are required for NPM1’s participation in HR. Generation of DNA double strand breaks (DSBs) results in pThr199 NPM1 recruitment to the DSBs and its co-localization with γH2AX [12,14,18,19]. This is a consequence of binding to K63-linked ubiquitinated histones that surround DSBs, via NPM1’s pThr199 domain that is comprised of an acidic tract and adjacent ubiquitin interacting motif–like domain [18]. Although earlier RNAi approaches [18,20] did not uncover a relationship between NPM1 and RAD51, a meticulous and thorough investigation by Xu et al. [14] utilizing multiple approaches revealed that SUMOylated K263 NPM binds to RAD51 and this association is required for RAD51 loading onto resected DNA at the DSB.

YTR107 is a substituted 5-((*N*-benzyl-1*H*-indol-3-yl)methylene)pyrimidine-2,4,6(1*H*,3*H*,5*H*) trione that inhibits pT199 NPM1 recruitment to DNA DSBs, co-localization with γH2AX foci and repair of DNA DSBs. YTR107 has been shown to radiosensitize seven NSCLC cell lines, breast carcinoma cells, colorectal adenocarcinoma cells, glioblastoma cells, and pancreatic carcinoma cells [12,19]. It was developed using a forward chemical genetics approach coupled with functional phenotypic screening for structure-activity relationships [12,21,22]. MS/MS pro-teomics and immunoblotting analyses of endogenous and purified recombinant NPM1 confirmed that NPM1 is YTR107’s molecular target and that YTR107 physically interacts with NPM1 [12]. Previously, we expressed recombinant His-tagged NPM1 (full length or aa 1–122) from a pET28a vector, purified the proteins and added them to biotinylated-YTR107-streptavidin magnetic beads. Extensive washing removed unbound protein. Immunoblotting demonstrated that both full length and truncated NPM1 bound directly to YTR107 [12]. Next, we undertook a virtual docking analysis of the YTR107/NPM1 binding interface [22]. The analysis revealed that the top scoring docked poses of YTR107 binding were with monomeric NPM1 and were located at the center of the interface-forming surfaces of NPM1 that interact with other monomeric NPM1 units in the assembly of the functional pentameric form of NPM1. This suggests that YTR107 likely disrupts oligomerization of NPM1.

Here we show that YTR107 inhibits DNA DSB-induced RAD51-NPM1 interactions and RAD51 foci formation, but does not inhibit γH2AX, pMDC1, or 53BP1 foci formation. We found that YTR107 and the PARP1/2 inhibitor ABT-888 function synergistically. YTR107 caused significant radiosensitization of tumor TICs, consistent with the knowledge that RAD51 can contribute to TIC intrinsic radiation resistance [23]. Adding YTR107 to a fractionated irradiation regiment (2.2 Gy q.d. x 7 days) diminished tumor growth and increased overall survival of A549 NSCLC tumor-bearing mice. Taken all together the data indicate that YTR107 can be an effective radiosensitizing strategy for NSCLC cells with varying genomic landscapes.

## 2. Materials and methods

A complete and comprehensive description of reagents and methods can be found in Supplemental Information.

### 2.1. Tumor initiating cell assay: xenograft growth

These studies were approved by the Vanderbilt University School of Medicine Institutional Animal Care and Use Committee and performed under guidelines outlined in The Guide for the Care and Use of Laboratory Animals. Tumor cell monolayers (5 × 10^6^ cells) were exposed to DMSO or 35 μM YTR107 for 30 min prior to, during sham treatment or irradiation (5 Gy, ^137^Cesium @ 2 Gy/min) and for 90 min afterwards. Cells were trypsinized, diluted to the indicated numbers, mixed with Matrigel and injected S.C. (100 μl) into the flanks of 8 to 10-week-old athymic Nude Fox1n1n female mice (Envigo). Tumor growth was calculated 2x per week according to the formula: length x (width)^2^/2). Forty days after tumor cell injection mice were categorized as tumor-bearing or no presence of tumor.

### 2.2. Tumor growth following fractionated X-irradiation to xenografts

These studies were approved by the Vanderbilt University School of Medicine Institutional Animal Care and Use Committee and performed under guidelines outlined in The Guide for the Care and Use of Laboratory Animals. Hindlimbs of 8 to 10-week-old athymic Nude Fox1n1n female mice (Envigo) were subcutaneously implanted with 5 × 10^5^ A549 tumor cells. When tumors were palpable mice were randomized to the following protocols: 7 i.p. injections of (a) DMSO (25 μl) or (b) YTR107 (20 mg/kg) in DMSO (25 μl). Sixty min after injection tumors were administered (c) 0 Gy or (d) 2.2 Gy of x-rays (300 kVp/10 mA, 2 Gy/min). Mice were shielded such that only the tumors were irradiated. Digital calipers were used to obtain the length and width of each tumor. Tumor volume was calculated 2 times per week, according to the formula: length x (width)^2^/2).

### 2.3. Statistical analysis

GraphPad Prism Version 8.4.3 was used to perform all statistical analyses. Non-parametric Mann-Whitney U tests with Welch’s correction were used to analyze differences between groups. The survival of tumor-bearing mice was analyzed by Kaplan Meier. *P* < 0.05 was considered statistically significant.

## 3. Results

### 3.1. YTR107 inhibits the association of NPM1 with RAD51 in irradiated cells

Human embryonic kidney 293 (HEK293) cells are derived from a human primary embryonic kidney cell culture and express adenovirus type 5 E1A E1B gene products [24,25]. Stepanenko and Dmitrenko [26] performed an extensive literature review and concluded that HEK293 cells have the ability to form colonies in soft agar and tumors when injected into immunocompromised mice.

**Fig. 1.**
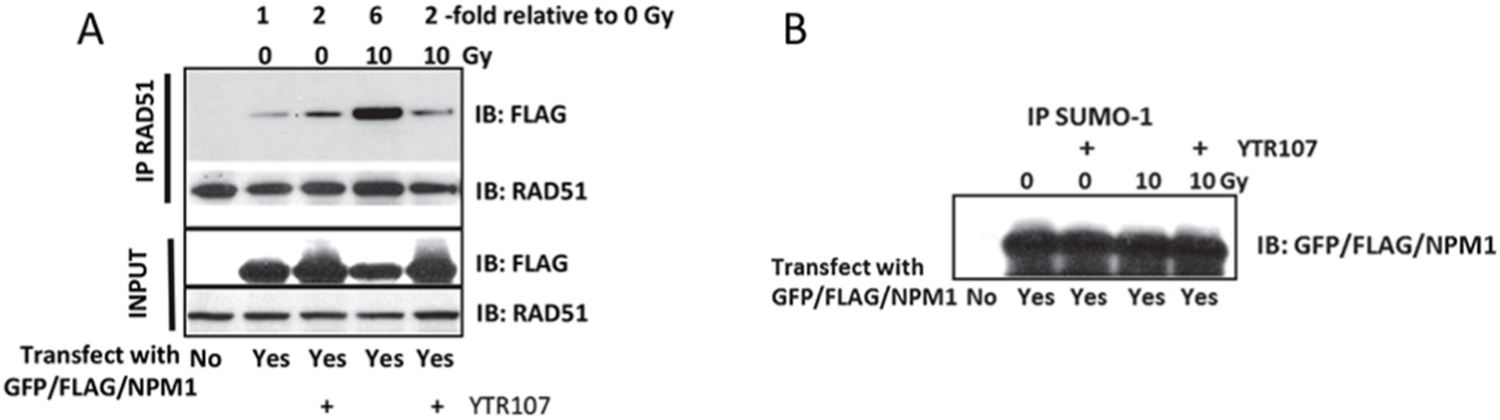
YTR107 inhibits NPM1/RAD51 interactions. A) HEK293 cells were transfected with a plasmid that expressed a chimeric GFP/FLAG/NPM1 protein. Five days after transfection the cells were exposed to 0 or 35 μM YTR107 for 30 min prior to administering 0 or 10 Gy. Two hrs later cells were solubilized in 0.5% NP40 buffer. Protein was immunoprecipitated using a RAD51 antibody. Immunoprecipitated protein was immunoblotted with FLAG antibody. The expression of immunoprecipitated GFP/FLAG/NPM1 relative to immunoprecipitated RAD51 is shown at the top of figure. B) HEK293 cells were transfected with a plasmid that expressed a chimeric GFP/FLAG/NPM1 protein. Five days after transfection the cells were exposed to 0 or 25 μM YTR107 for 30 min prior to administering 0 or 10 Gy. Two hrs later cells were solubilized in 0.5% NP40 buffer. Protein was immunoprecipitated using a SUMO-1 antibody. Immunoprecipitated protein was immunoblotted with FLAG antibody.

HEK293 cells were transiently transfected with a GFP/FLAG/NPM1 expression vector. Five days after transfection cells were exposed to 0 or 35 μM YTR107 for 30 min, then administered 0 or 10 Gy. Two hrs later cells were solubilized. Solubilized protein lysate was immunoprecipitated with a RAD51 antibody or an antibody to SUMO1. Immunoprecipitated protein was immunoblotted with FLAG antibody. In unirradiated cells immunoprecipitation with the RAD51 antibody yielded little NPM1 (Fig. 1A). The increase in NPM1/RAD51 association in unirradiated cells exposed to YTR107 can be attributed to DNA damage resulting from YTR107-mediated replication stress [12]. DNA damage produced by 10 Gy increased the association of NPM1 with RAD51 6-fold (Fig. 1A), consistent with the results reported by Xu et al., [14]. However, exposure to YTR107 inhibited the interaction of NPM1 with RAD51 (Fig. 1A).

NPM1 is constitutively SUMOylated by SUMO1–3 isoforms at K230 and K263, the latter site by p14^ARF^ [17,27]. SUMOylation at K263 is required for NPM1 binding to RAD51 [14]. As stated above, cell lysate was also immunoprecipitated with an antibody to SUMO1 in order to rule out the possibility that YTR107 affected constitutive SUMOylation of NPM1. This approach for investigating NPM1 SUMOylation is similar to the approach used by Tago et al. [17]. As shown in Fig. 1B, recovery of FLAG immunoreactive protein was independent of YTR107 exposure in unirradiated and irradiated cells. Thus, exposure to YTR107 did not significantly diminish NPM1 SUMOylation.

### 3.2. YTR107 inhibits RAD51 foci formation

**Fig. 2.**
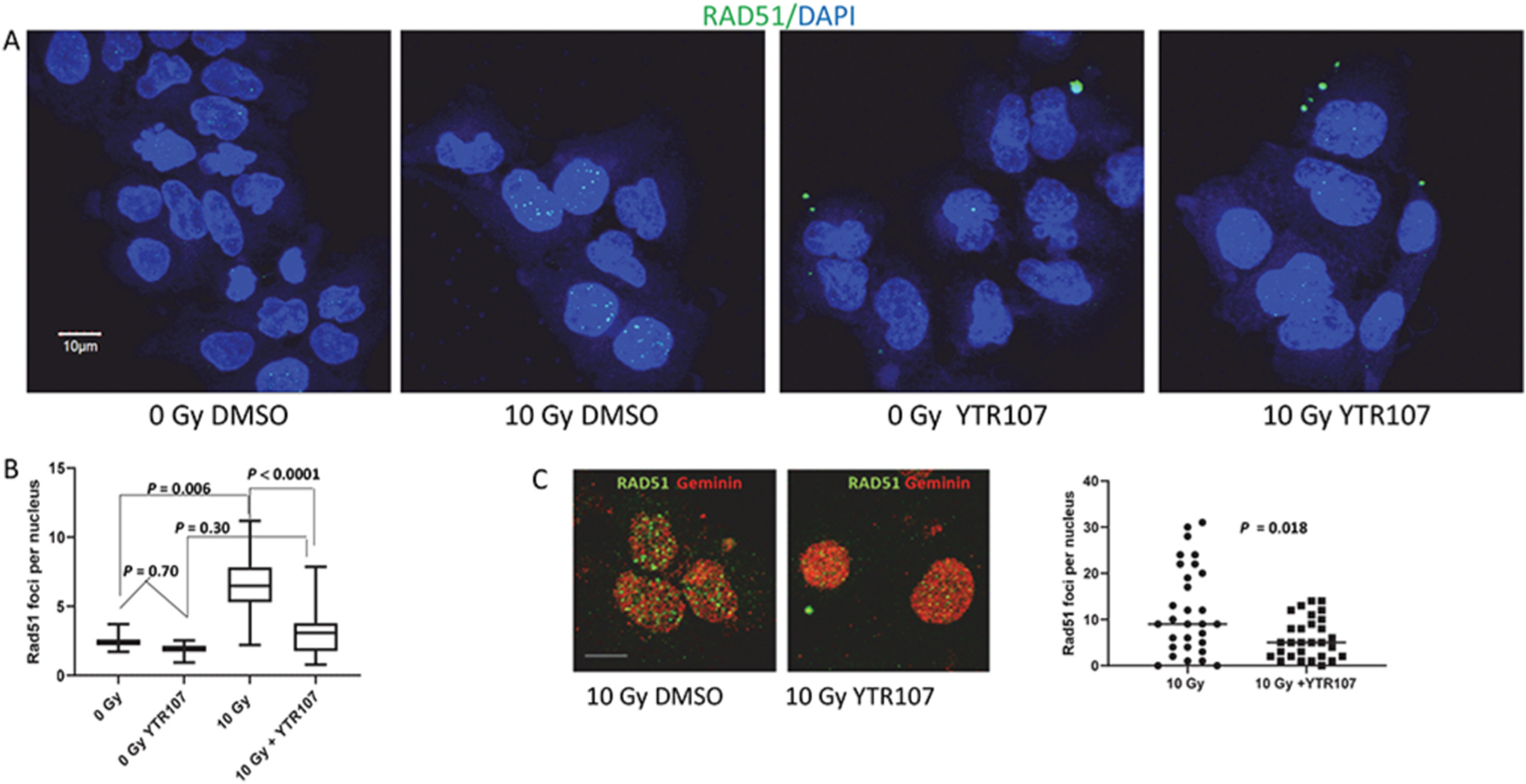
RAD51 foci formation is inhibited by YTR107. (A) A549 cells were exposed to 25 μM YTR107 for 30 min prior to, during and for 4 h after 0 or 10 Gy. Cells were immunostained for RAD51 and DAPI counterstained. (B) Quantification of individual foci per nuclei is expressed as box and whisker plots, max to min, and represents the average of four independent experiments. N = 132 for 0 Gy DMSO; N = 109 for 0 Gy YTR107; N = 365 for 10 Gy (DMSO); N = 208 for 10 Gy YTR107. (C) Cells were immunostained for RAD 51 and Geminin, a S/G2 phase marker. (D) Quantification of individual RAD51 foci per nuclei in Geminin expressing cells. white line = 10 μm.

As YTR107 has been shown to inhibit NPM recruitment to DNA DSBs [12,18,19] and YTR107 inhibits the association of NPM1 with RAD51 (Fig. 1), it was of interest to determine if YTR107 would inhibit RAD51 foci formation in irradiated cells. As illustrated in Fig. 2A and B radiation-induced RAD51 foci formation was reduced 2-fold when cells were irradiated in the presence of YTR107 (*P* < 0.0001). Fig. 2C illustrates YTR107-mediated suppression of RAD51 immunostaining in Geminin expressing cells. Geminin is expressed in S and G2 phases of the cell cycle and interacts with Cdt1 to prevent re-replication during S phase [28], thus confirming that YTR107 inhibits RAD51 foci formation in S/G2 cells. Used for their excellent optical properties, studies of HeLa cells confirm that YTR107 inhibits radiation-induced RAD51 foci formation (Supplemental Fig 1). YTR107-mediated inhibition was specific for RAD51, as it did not inhibit radiation-induced γH2AX foci formation, pMDC1 foci formation or 53BP1 foci formation (Supplemental Fig 2).

### 3.3. Synergy between YTR107 and the PARP1/2 inhibitor ABT 888

Simultaneous inhibition of RAD51 and PARP1, using small molecule inhibitors, has been shown to synergistically sensitize cells to DNA damaging agents [29]. Therefore, it was of interest to determine whether YTR107 and PARP inhibition would function synergistically in irradiated cells where each Gy of photons produces approximately 35 DSBs [30]. It is important to note that YTR107 does not inhibit PARP activity [12]. The question of synergy was addressed using irradiated (4 Gy) A549 cells exposed to the PARP1/2 inhibitor ABT 888 (10 μM for 72 h), YTR107 (25 μM for 2 h), or a combination of both compounds (Table 1). A Radiation Enhancement Ratio (RER) calculation [31] yielded a value of 7.67 (0.33/0.043), indicating that combining YTR107 and ABT 888 in irradiated cells produced a synergistic effect. The RER quantifies enhancement of DNA damage on an isodose basis (ie, 4 Gy). Additionally, the data were analyzed using CompuSyn software [32] and a combination index (CI) of less than 1 was derived (Table 1), again indicating synergism when irradiated cells were exposed to a combination of YTR107 and ABT 888.

Previously, we reported that exposing HT29 colon carcinoma cells to YTR107 alone induced RPA32 filament formation, CHK1 phosphorylation at S317, γH2AX foci formation, and accumulation of cells at the G2 check point, all characteristics of replication stress [33]. Furthermore, persistent YTR107-induced replication stress resulted in a loss of cell viability. Replication stress results in replication fork uncoupling that leads to extended single strand DNA regions that are coated with RPA [34]. If the single strand regions exceed a critical length, then RAD51 partially replaces the RPA. RAD51 stabilization and fork reversal requires PARP activation [34]. PARylation and p21 work together to monitor DNA fork progression [33]. PARP1 inhibition can result in accelerated fork progression, replication stress and reduced cell viability [33]. Given that YTR107 induces a cytotoxic replication stress we determined whether PARP1 activity was required for resolving the stress.

Hydroxyurea (HU) is a well characterized agent for inducing replication stress and this can be monitored by RPA70 coating single strand DNA filaments [35], as illustrated in Supplemental Fig 3A where A549 cells were exposed to 3 mM HU for 24 h. Exposing A549 cells to YTR107 (25 μM/5 h) also induces RPA70 filament formation, a consequence of replication stress (Supplemental Fig 3A). The mass-action software program, CompuSyn [32] was used to determine the relationship between ABT 888 and YTR107-mediated cytotoxicity, measured by colony formation (Supplemental Fig 3B). A combination index (CI) plot (Supplemental Fig. 3C) derived from the relationship between drug dose and effect (1-surviving fraction, Supplemental Fig. 3B) indicates that CI values are all less than 1.0. Thus, the synergism presented in Table 1 and Supplemental Fig. 3C are interpreted to be a consequence of YTR107-mediated inhibition of RAD51.

### 3.4. YTR107 inhibits repair of DNA DSBs

**Fig. 3.**
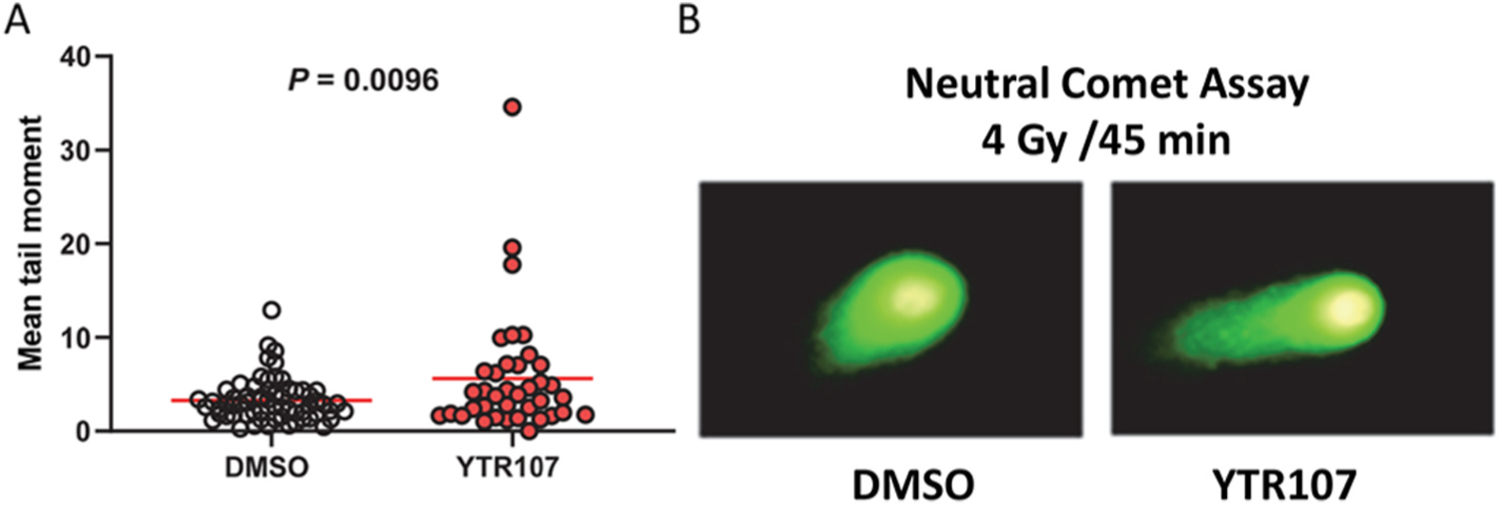
YTR107 inhibits repair of DSBs. H460 cells were exposed to 25 μM YTR107 for 30 min prior to, during administration of 4 Gy and for 45 min after γ-irradiation. Cells were then subjected to neutral comet assays. (A) Quantification of mean tail moment. Red bar = mean. (B) Representative comet images.

Inhibition of RAD51 foci formation would be expected to negatively impact repair of DNA DSBs. Neutral comet assays were used to address this expectation. The assays were conducted using H460 cells, which provide excellent optical properties in this assay. Comet moment is a well characterized measure of DNA DSBs [36]. H460 cells were administered 4 Gy in the absence or presence of YTR107. Cells were allowed 4 h to repair DNA damage and then subjected to the neutral comet assay. Exposure to YTR107 during the repair period resulted in a larger tail moment (1.7-fold compared to irradiation in DMSO, *P =* 0.0096), indicative of a greater number of unrepaired DNA DSBs compared to cells irradiated in DMSO, Fig. 3.

### 3.5. YTR107 increases radiation sensitivity

**Fig. 4.**
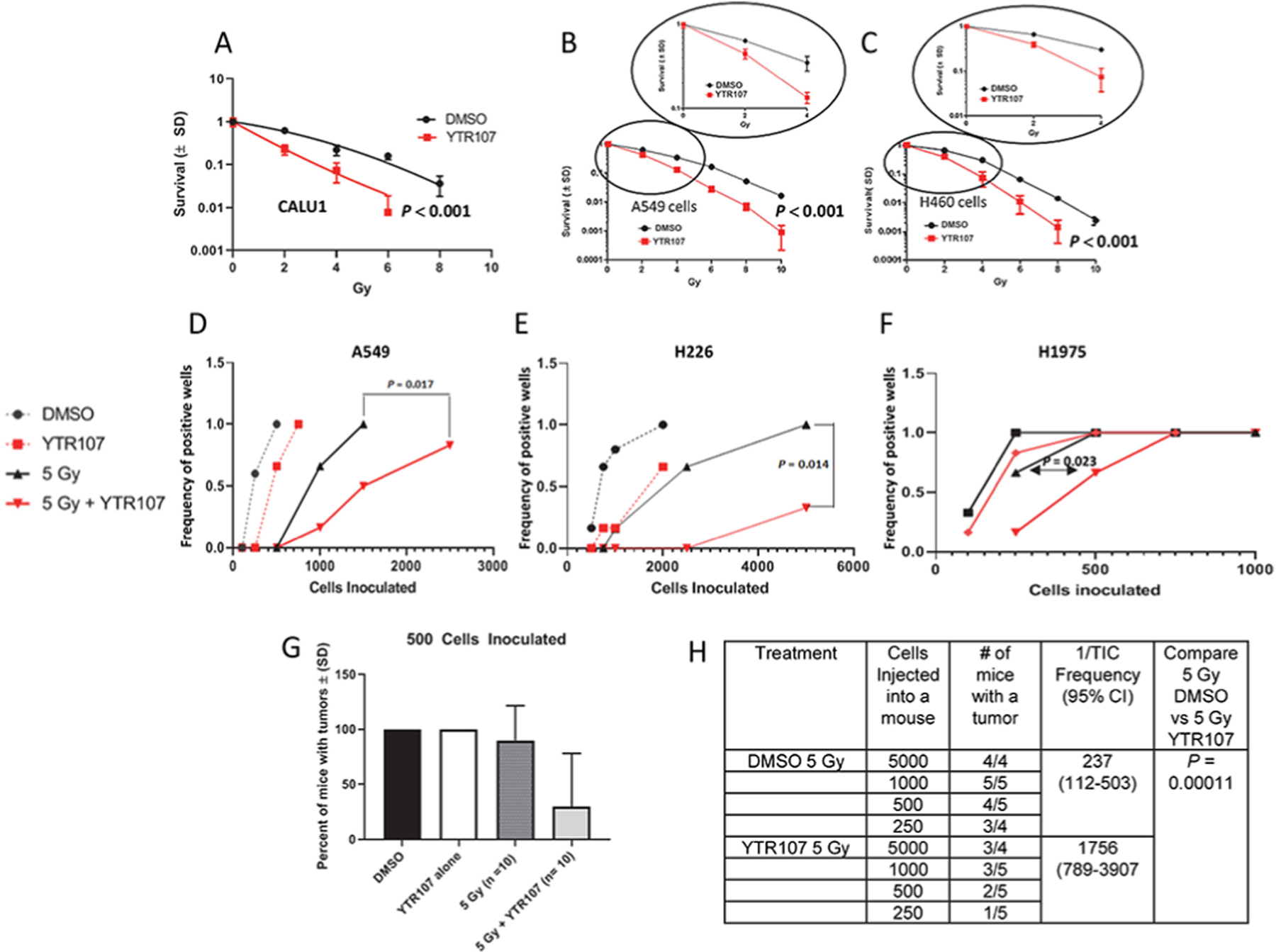
YTR107-mediated radiosensitization. Calu1 (A), A549 (B), and H460 (C) cells were exposed to YTR107 for 30 min prior to, during and or only 90 min after irradiation. Clonal survival to YTR107 alone was: 38%, 72% and 59% for Calu1, A549, and H460 respectively. Colony formation assays were used to generate the survival curves fitted by least squares to equation S = e^−αD−βD^2^. The dose response curves shown in the circles illustrates survival as a function of dose up to 4 Gy. (D–F) For tumorsphere assays A549, H226, and H1975 cells were exposed to YTR107 (35 μM) or DMSO for 30 min prior to, during and for 90 min after irradiation (5 Gy). After extensive washing, varying cell numbers were inoculated into ultra-low adhesion wells for tumorsphere limiting dilution assays. (G & H) Monolayers (5 × 10^6^ cells) of A549 were exposed to DMSO, the solvent control, or YTR107 (35 μM) for 30 min before, during and for 90 min after 0 or 5 Gy and then washed extensively. Then 500 washed cells (G) or varying numbers of washed cells (H) were injected into the flanks of athymic nude mice. Tumor formation was monitored for 40 days. TIC – Tumor Initiating Cells, *P* value compares 1/TIC Frequency for 5 Gy alone + DMSO vs 5 Gy + YTR107.

Calu1, a lung epidermoid carcinoma cell line, A549, a lung adenocarcinoma cell line, and H460, a large cell lung cancer cell line, were exposed to YTR107 and irradiated (Fig. 4A–C). The mutation status of each cell line is shown in Supplemental Table 1. Survival data was fitted by least squares to equation S = e^−αD−βD^2^. For each cell line the presence of YTR107 during and for only 90 min after irradiation produced a statistically significant increase in radiation sensitivity. The dose reduction factor, determined at 10% survival was 1.8, 1.6, and 1.5 for Calu1, A549, and H460 cells, respectively, *P* < 0.001. Importantly, at the clinically relevant dose of 2 Gy, YTR107 reduced survival by 63% (Calu1), 30% (A549) and 39% (H460), (*P* < 0.001). These changes following a single dose of 2 Gy can be fully appreciated by the knowledge that radiation therapy is normally administered in 60 or more 2 Gy fractions. In contrast, YTR107 did not radiosensitize non-tumorigenic mouse embryo fibroblasts that were exposed to 50 μM YTR107 or DMSO for 30 min before, during and 90 min after 6 Gy irradiation, which induces approximately 210 DSBs. We used high radiation and drug dosing to increase the stringency of this test. There were no differences (*P* > 0.05) in plating efficiency nor survival fraction for YTR107 + 6 Gy (0.42 ± 0.09) vs. DMSO +6 Gy (0.43 ± 0.04), by colony formation assay (Supplemental Fig 4). This result corroborates previously published data in which it was shown that YTR107 did not inhibit recruitment of NPM1 to radiation-induced damaged chromatin in human IMR-90 lung fibroblasts but did inhibit NPM1 recruitment in A549 and Calu1 cells [37].

The ability of single tumor cells to grow and proliferate as spheroids in serum-free, non-adherent culture conditions is an accepted assay to estimate the percentage (frequency) of tumor initiating cells (TICs) present in a population of tumor cell [38]. We exposed A549 cells, H226 (squamous mesothelioma) cells and H1975, lung adenocarcinoma cell line, to YTR107 + irradiation (5 Gy). After extensive washing, varying cell numbers were inoculated into serum-free, stem cell medium and plated into ultra-low adhesion culture plates that promote spheroid formation. Irradiation in the presence of YTR107 produced significant radiosensitization in all 3 TIC populations (Fig. 4D–F), consistent with the knowledge that TIC radiation resistance can be modulated by RAD51 activity [39]. Next, we investigated the ability of YTR107-sensitized cells to form tumors in athymic mice. A549 monolayers were irradiated with 0 or 5 Gy in the presence of YTR107 or DMSO. Ninety min after irradiation or sham treatment athymic female mice were inoculated with 500 extensively washed cells and tumor growth followed for 40 days (Fig. 4G). Tumors formed in 100% of mice injected with unirradiated cells treated with either DMSO (N = 5) or YTR107 (N = 5). Additionally, tumor growth was observed in 9 of 10 mice injected with DMSO-treated cells administered 5 Gy but in only 3 out of 9 mice injected with YTR107-treated cells administered 5 Gy (*P =* 0.02). A limiting dilution analysis [40,41] was performed. A549 monolayers were irradiated with 5 Gy while exposed to YTR107 or DMSO. Ninety min later cells were washed and athymic female mice were then inoculated with varying numbers of cells (Fig. 4H). Tumor growth was monitored for 40 days. The frequency of tumor formation was used to calculated 1/TIC [40]. The 1/TIC frequency was 1756 (95%C.I. = 789–3907) for YTR107 + 5-Gy treated cells. This is 7.4-fold greater (*P* = 0.000117) than 237 (95%C.I. 112–503) for DMSO + 5-Gy treated cells. Thus, combining YTR107 with radiation significantly increased (7.4-fold) the number of cells needed to form a tumor (vs. radiation alone).

**Fig. 5.**
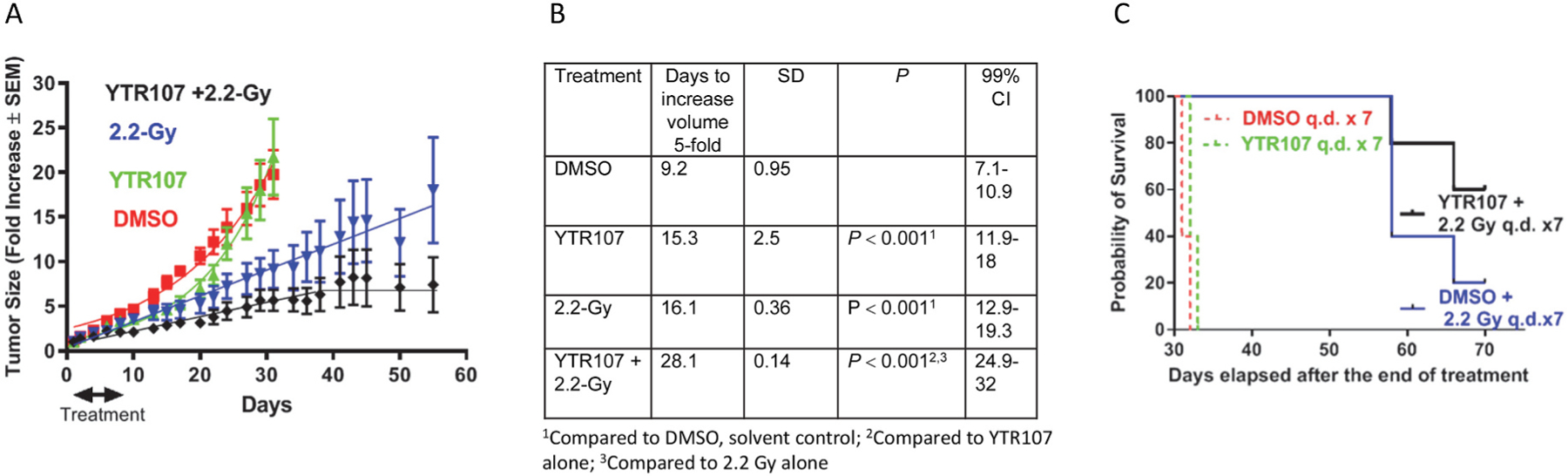
NSCLC xenograft growth delay is enhanced by YTR107. A549 xenograft-bearing mice were subjected to a 7-day regimen consisting of YTR107 (20 mg/kg) or solvent control (DMSO) administered IP 1 h before tumors received 0 or 2.2-Gy. Tumor growth curves are shown in panel (A). Days (±SD) required to achieve a 5-fold increase tumor volume are plotted in (B). (C) Kaplan Meier survival analysis. A549 xenograft-bearing mice were subjected to a 7-day regimen consisting of YTR107 (20 mg/kg) or solvent control (DMSO) administered IP 1 h before tumors received 0 or 2.2-Gy.

We tested the ability of YTR107 to enhance fractionated radiation therapy (Fig. 5A). A549 tumor-bearing mice received a regimen consisting of YTR107 (20 mg/kg/day IP or DMSO, IP) given 1-hr before tumor irradiation (2.2-Gy) or sham treatment (0 Gy). Tumor-bearing mice were treated once a day from Monday through Friday, not treated on Saturday or Sunday, and then treated the next Monday and Tuesday (Fig. 5A). In vehicle-treated mice unirradiated tumors took 9.2 days to increase volume 5-fold whereas 15.3 days (an increase of 66%) were required to achieve the same tumor growth in YTR107-treated mice (0 Gy, *P =* 0.001, Fig. 5B). One interpretation is that the YTR107-mediated tumor growth delay in unirradiated tumors is due to replication stress induced by this drug.

Irradiated tumors (2.2 Gy + DMSO) required 16.1 days to increase volume 5-fold, which was 6.9 days more than required for unirradiated, DMSO-treated tumors (*P =* 0.001, Fig. 5B). 28.1 days were required for tumors treated with YTR107 and 2.2 Gy to increase volume 5-fold, (a 74.5% increase over the tumor growth delay produced by 2.2 Gy alone (*P =* 0.001, Fig. 5B). Supplemental Figure 5 illustrates the growth rate for individual tumors by treatment group and mouse weights. Inspection of the individual tumor growth curves for irradiated tumors (±YTR107) indicates a heterogenous response to irradiation (Supplemental Fig. 5C and 5D). Tumor growth delay heterogeneity does not appear to be a consequence of differences in tumor size at the initiation of treatment. For the treatment group 2.2 Gy + DMSO the mean tumor volume was 59 (±14 SD) mm^3^ and for tumors treated with 2.2 Gy + YTR107 the average tumor size was 53 (±6 SD) mm^3^, *P* > 0.05. Body weight in gm was independent of treatment (Supplemental Figure 5E).

Kaplan Meier analyses of survival is shown in Fig. 5C, calculated over a 70-day interval. The median survival for tumor-bearing mice treated with 7-fractions of DMSO and 2.2-Gy was of 58 days. In contrast, median survival had not been reached at 70 days (study termination) for tumor-bearing mice treated with YTR107 + 2.2-Gy. The Cox proportional log rank hazard ratio was 0.24 (*P =* 0.0034) comparing irradiated (+DMSO) vs. unirradiated (+DMSO) tumor-bearing mice. It was 0.17 (*P =* 0.0023) comparing YTR107 + 2.2-Gy irradiation vs. no irradiation (0-Gy ± YTR107). Addition of YTR107 to the seven 2.2 Gy fractions lowered the hazard ratio by 29%.

## 4. Discussion

Nucleophosmin1 (NPM1) is a pentameric [42] chaperone. It undergoes liquid-liquid phase separation that contributes to the liquid-like structure of nucleoli [43] and forms immobilized scaffolds with other molecules, eg p14ARF [44]. NPM1 has crucial roles in DDR pathways [13–15], but it is unknown whether NPM1’s phase separation attributes are required for its involvement in the DDR, as is the case for 53BP1 [45].

NPM1 accumulates very rapidly at sites of DNA DSBs [14]. Recruitment and co-localization with γH2AX at sites of DNA DSBs requires NPM1 phosphorylation at Thr199 [12,14,18,19] and K63-linked ubiquitinated histones generated by RNF8 and RNF168 [18]. Previously, we have shown that YTR107 inhibits NPM1 accumulation at sites of DNA DSBs and its co-localization with γH2AX foci [12,19] and does so without affecting the phosphorylation status of Thr199 [19]. SUMOylation at K263 NPM1 by Arf [17] licenses NPM1 binding to RAP80/BRCA1-A complexes and subsequent recruitment of the BRCA1-A complex to DNA DSBs [14]. Additionally, SUMOylated K263 NPM binds to RAD51, promoting RAD51 loading onto DNA filaments [14]. Loss of NPM1 or failure to SUMOylate K263 NPM1 diminishes HR, as measured by the HR repair plasmid DR-GFP [14]. We found that YTR107 blocks SUMOylated NPM1 from associating with RAD51 in irradiated cells but does not result in deSUMOylation. Our data indicate that YTR107 targets NPM1, inhibiting its association with RAD51 and subsequent recruitment to sites of DNA damage.

The crystal structure of the human NPM1 core has been solved [46], with the results indicating that NPM1 monomers fold into an 8-stranded β of jellyroll topology, forming a single domain. Monomers are organized into pentamers, with the β-strands aligned approximately parallel to the fivefold axis [46]. A virtual docking analysis of YTR107 with NPM1 [22] supports the hypothesis that YTR107’s mode of action is to prevent NPM1 pentameric formation and that it is this oligomeric form that is required for participation in HR. The knowledge that the small molecule NSC348884 disrupts NPM1 oligomer formation [19,47] and radiosensitizes [19] supports this hypothesis. However, formal testing of this hypothesis is beyond the scope of this current investigation.

We show that YTR107-mediated inhibition of NPM1 impairs RAD51 recruitment to sites of DNA damage in irradiated cells and leads to radiosensitization of three NSCLC cell lines with various mutational burdens (Fig. 4A–C). Previously, we have shown that YTR107 radiosensitizes seven NSCLC cell lines, breast carcinoma cells, colorectal adenocarcinoma cells, glioblastoma cells, and pancreatic carcinoma cells [12]. These data suggest that targeting of NPM1 can be an effective radiosensitizing strategy for cells with varying genomic landscapes. To what do we attribute YTR107’s radiosensitizing properties? Based on the knowledge that YTR107 does not radiosensitize NPM1 deficient cells [12,19], it may be concluded that YTR107-mediated radiation sensitization is a consequence of targeting NPM1. NPM1 has been shown to promote APE1 activity, which if inhibited can result in radiation sensitization [48–50]. NPM1 also promotes RAD51 recruitment to single-stranded DNA during repair of DSBs [14], and inhibition of RAD51 [29] results in radiosensitization [39]. Consistent with this knowledge we found that the PARP inhibitor ABT 888 and YTR107 acted synergistically to radiosensitize cells (Table 1). While we cannot rule out other mechanisms, these data support a hypothesis that YTR107-mediated radiosensization is in part a consequence of inhibition of APE1 and RAD51.

Tolerance to oncogene-induced replication stress requires activation of the DDR [51,52]. However, sustained inhibition of critical elements of the DDR can lead to replication stress generation of one ended double strand breaks and cell lethality [34,53–55]. RAD51 plays an integral role in the response to replication stress by promoting fork reversal and by suppressing formation of DNA double strand breaks [56]. YTR107 induces a replication stress [ref [12] and Supplemental Fig 3]. Addition of the PARP inhibitor ABT 888 to cells exposed to YTR107 synergistically enhanced replication-induced cell death (Supplemental Fig. 3B and 3C). One interpretation of this synergism is that NPM1 is required for tolerance to replication stress. This conclusion is based on NPM1’s role in HR for recruiting RAD51 to form a nucleoprotein filament on single stranded DNA, the importance of RAD51 recruitment to single-stranded DNA during replication stress [57] and PARP1’s role in resolution of stalled replication forks [58]. As replication stress has been shown to synergize with ionizing radiation-induced DNA DSBs to increase radiosensitization [59], we conclude that YTR107-mediated radiosensitization is a consequence of replication stress-induced DNA damage and inhibition of radiation-induced DSBs.

The failure of radiation therapy to control tumor growth can be attributed, in part, to the presence of intrinsically radioresistant cancer stem cells. Tumor-initiating cells (TICs) are considered a surrogate for cancer stem cells and TIC radiosensitization can be monitored using tumorsphere and xenograft assays. In the tumorsphere assays exposure to YTR107 produced significant radiosensitization in all three tumor cell lines tested (Fig. 4D–F). When A549 cells were irradiated (5 Gy) *in vitro* and then various numbers of cells injected into athymic mice, an ELDA software analysis of the results [40] indicated a 1/TIC frequency of 237 (112–503, 95% CI, Fig. 4H). In contrast, exposure to YTR107 for only 30 min prior to, during and for 90 min after administration of 5 Gy increased the 1/TIC frequency to 1756 (789–3908, 95% CI), a 7.4-fold increase (*P =* 0.000117). These results indicate that YTR107 radio-sensitizes TICs.

Tumor-bearing mice were injected i.p. with YTR107 or DMSO as solvent control, followed 1 h later by administration of 2.2 Gy to the tumors q.d. x 7. Administration of YTR107 plus 2.2 Gy in 7 fractions significantly inhibited tumor progression (74.5%). Log rank hazard ratio, when measured 70 days after the final treatment, was decreased 29% by the addition of YTR107 to the irradiation protocol. The magnitude is a reflection, in part, 1) of the fact that RAD51 participates in the repair of 15–20% of induced DSBs in S and G2 cells [60] and 2) the heterogeneity of tumor response. It is also important to recognize that definitive fractionated radiation therapy for stage III NSCLC is delivered in thirty to thirty-five 2 Gy fractions [61]. Thus, 7 fractions represent 20–23% of the total number of therapeutic fractions administered. One could hypothesize that administration of YTR107 throughout a full course of radiation therapy would significantly improve therapeutic outcome. Taken all together these data support the concept that YTR107 combined with radiation therapy may be an effective strategy for controlling advanced NSCLC.

## Supplementary Material

Supplemental Information & Supp.Tab 1& Supp.Fig Legends

Supp.Fig1

Supp.Fig2

Supp.Fig3

Supp.Fi4

Supp.Fig5

## Figures and Tables

**Table 1:** Radiosensitization by YTR107 and ABT 888.

Treatment	Survival (±SD)	Radiation Enhancement Ratio	P Value	Fa	Combination Index
None	1.0^a^ (0.10)				
YTR107 alone	0.48^a^ (±0.08)				
ABT 888 alone	0.83^a^ (±0.09)				
YTR107+ ABT 888	0.24^a^ (±0.10)				
4 Gy	0.33^a^ (±0.03)			0.67	
YTR107 +4 Gy	0.19^2^ (±0.03)			0.81	
ABT888 +4 Gy	0.14^2^ (±0.02)			0.86	
YTR107+ ABT888 +4 Gy	0.043^2^ (±0.01)	7.67	*P <* 0.01	0.96	0.00029

A549 cells in full growth medium (10% FBS) were exposed to YTR107 (25 μM), ABT888 (10 μM) or both YTR107 and ABT888 for 30 min prior to, during irradiation (4 Gy), and for 90 min after irradiation. Cells were washed extensively, incubated in full growth medium supplemented with ABT888 (10 μM) for 70 h, washed again, and allowed to form colonies (>50 cells per colony) in full growth medium. The data presented in the Table represent results averaged from two experiments.

a Survival corrected for plating efficiency.

b Survival corrected for cell death induced by drug treatment. Radiation Enhancement Ratio is defined in ref 33. CI = combination index, analyzed using CompuSyn software [32].
