# Supplementary material for: Targeting NPM1 in irradiated cells inhibits NPM1 binding to RAD51, RAD51 foci formation and radiosensitizes NSCLC
Source: Cancer Lett. Author manuscript; Available in PMC 2021 Mar 1. (PMC7822076; doi:10.1016/j.canlet.2020.12.023)
Supplement: Supplemental Information & Supp.Tab 1& Supp.Fig Legends [file NIHMS1658200-supplement-Supplemental_Information____Supp_Tab_1__Supp_Fig_Legends.docx]

**Supplemental Information**

**Materials and Methods:**

**Plasmids and Transfection**

The GFP/FLAG/NPM1 expression plasmid was obtained from Addgene (#17578) and transiently transfected into HEK293 cells. Cells were administered 0 or 10 Gy five days after transfection. Cells were solubilized 2 hrs after irradiation. Cell lysate was immunoprecipitated with a RAD51 antibody (mouse, Thermo MA5-14419 @ 1:100) or an antibody to SUMO1 (rabbit, Cell Signaling 4930 @ 1:50). Immunoprecipitate was immunoblotted with antibody to FLAG (rabbit, Thermo PA1-984B) and anti-Rabbit IgG-HRP (GE Healthcare NA934), then stripped and reblotted with antibody to RAD51 (mouse, Thermo MA5-14419) and anti- Mouse IgG (Promega W402B).

**Cells Lines, Cell Culture, Tumorsphere Culture, and Colony Formation Assays**

HEK 293, A549, H226, H460, Calu1, H1975, and mouse embryo fibroblast cell lines were obtained from ATCC and were free from *Mycoplasma* (Mycoplasma detection kit, Biotool #B39032). Cells were maintained in ATCC recommended medium supplemented with 10% fetal bovine serum at 37°C in 5% CO_2_ humidified incubators.

For tumorsphere formation assays monolayers of cells (70% confluent) were treated with 0 or 35 μM YTR107 for 30 min, irradiated using at 5 Gy using a ^137^Cs Mark 1 irradiator (2 Gy/min), incubated at 37°C for 90 min, washed extensively, trypsinized and various numbers of cells inoculated into 24-well ultra-low attachment plates containing 1x N2 supplement (Thermo Fisher Scientific), 20ng/ml hEGF (PeproTech AF-100-15), and 20ng/ml hFGF-basic (PeproTech 100-18B) in RPMI 1640. Tumorsphere formation (clonogenicity) was quantified 3 weeks after inoculation.

Colony formation assays were performed according to (Ref 14). Cells were exposed to 0 or 25 μM YTR107 for 30 min prior to, during irradiation using a ^137^Cs Mark 1 irradiator (2 Gy/min) and for 90 min after irradiation. Cells were washed and colony formation quantified 14 days after irradiation. Colonies were defined as having more than 50 cells per colony. Dose reduction factors were derived from the ratio of γ-irradiation dose levels needed to reduce survival to 10% (Irradiation in vehicle control, DMSO/Irradiation in YTR107).

**Confocal Imaging of Immunofluorescent Stained Slides**

Cells attached to microscope cover slips were sham treated, exposed to hydroxurea, YTR107, or irradiated using a 137Cs Mark 1 irradiator (2 Gy/min). To visualize RPA70 foci cells were washed with 4°C cytoskeleton buffer, extracted with 0.5% Triton X-100 in CSK buffer and fixed (Dimitrova and Gilbert, Exp Cell Res, 254:321-327, 2000). Cells were immunostained with antibody to RPA70 (rabbit - Abcam ab79398). To visualize radiation-induced repair foci, fixed cells were immunostained with antibodies to: Rad51 (rabbit- Abcam ab133534), γH2AX (mouse-Millipore 05-636), pMDC1 (rabbit, phospho T4-Abcam ab35967) or 53BP1 (rabbit**-**Novus Bio. NB100-304). The following secondary antibodies were used: Donkey anti-rabbit 488 (Life Technologies, A21206), Donkey anti-mouse 568 (Life Technologies, A10037) and Donkey anti-mouse 488 (Life Technologies, A21202). Counterstaining was with DAPI. Confocal images of immunofluorescent staining were acquired using an Olympus FV-1000 inverted confocal microscope using a 60x oil immersion objective lens and a 1.5x optical zoom. Primary antibody-specific immunofluorescence intensity of radiation-induced repair foci per nuclei was quantified by ImageJ (NIH). Primary antibody-specific immunofluorescence immunoblot intensity was also quantified by ImageJ.

**Neutral Comet Assay**

Assays were performed using the Comet Assay kit following the manufacturer's instructions (Trevigen, # 4250-050-K).

| Cell Line | Mutations | TP53 Status |
| --- | --- | --- |
| Calu1, NSCLC | KRAS | Homozygous deletion |
| A549, NSCLC | KRAS & CDKN2A | wt |
| H460, NSCLC | KRAS & CDKN2A | wt |
| H1975, NSCLC | CKDN2A, PIK3CA, & EGFR | mutated |
| H226, squamous mesothelioma | CKDN2A | wt |

Supplemental Table 1 – Mutation Status of Cells

**Figure Legends for Supplemental Data**

Supplemental Figure 1. RAD51 foci formation is inhibited by YTR107. (A) HeLa cells were exposed to 25 μM YT107 for 30 min prior to, during and for 4 hrs after 0 or 10 Gy. Cells were immunostained for RAD51 and DAPI counterstained. (B) Quantification of individual foci per nuclei is expressed as box and whisker plots, max to min. The mean numbers of RAD51 foci per nucleus and total number of nuclei counted were: 3 (N = 44, DMSO + 0 Gy), 2 (N = 41, YTR107 + 0 Gy), 18 (N 33, DMSO + 2 Gy) and 4 (N = 34, YTR107 + 2 Gy. White line = 10 μm.

Supplemental Figure 2. Radiation-induced γH2AX, pMDC1 and 53BP1 foci formation is independent of YTR107. A549, Calu1, and H226 cells were exposed to 25 μM YTR107 for 30 prior to, during and for the indicated times after γ-irradiation. The dose administered and the time of fixation were adjusted to provide discrete foci optimized for quantification. Cells were immunostained for γH2AX foci (panel A), pMDC1 foci (panel B), or 53BP1 foci (panel C). Quantification of individual foci per nuclei is expressed as box and whisker plots, max to min. The number of nuclei counted per point is shown. White line = 20 μm.

Supplemental Figure 3. YTR107 and ABT 888 function synergistically to induce cell lethality. (A) A549 cells were exposed to 3 mM hydroxyurea (HU) for 24 hrs or to YTR107 (25 μM) for 5 hrs. Cells were immunostained for RPA70 foci formation. (B) A549 cells were treated with the indicated YTR107 concentrations for 5 hrs, the indicated ATB 888 concentrations for 72 hrs or a combination of YTR107 and ABT 888. Effect = 1- survival fraction. Surviving fraction determined by colony formation assays. (C) The mass-action software program, CompuSyn (Ref 32) was used to determine the relationship between ABT-888 and YTR107-mediated cytotoxicity.

Supplemental Figure 4. Survival of nontumorigenic mouse embryo fibroblasts after 6-Gy is unaffected by YTR107 exposure. Mouse embryo fibroblasts were exposed to 50 µM YTR107 or DMSO for 30 min before, during and 90 min after 6 Gy irradiation. Survival was assessed by colony formation assays, expressed as mean ± SD.

Supplemental Figure 5. YTR107 augments radiation-mediated tumor growth inhibition in mice bearing A549 xenografts. Tumor-bearing mice were subjected to a 7-day regimen consisting of (A & C) DMSO, solvent control or (B & D) YTR107 (20 mg/kg) administered i.p. 1 hr before tumors received 0 ( A & B) or 2.2-Gy (C & D). There was no difference in body weights among treatment groups (*P* > 0.05). Mean body weights (± SD) vs days are plotted in E.
