# Supplementary figures and images for: Targeting NPM1 in irradiated cells inhibits NPM1 binding to RAD51, RAD51 foci formation and radiosensitizes NSCLC
Source: Cancer Lett. Author manuscript; Available in PMC 2021 Mar 1. (PMC7822076; doi:10.1016/j.canlet.2020.12.023)

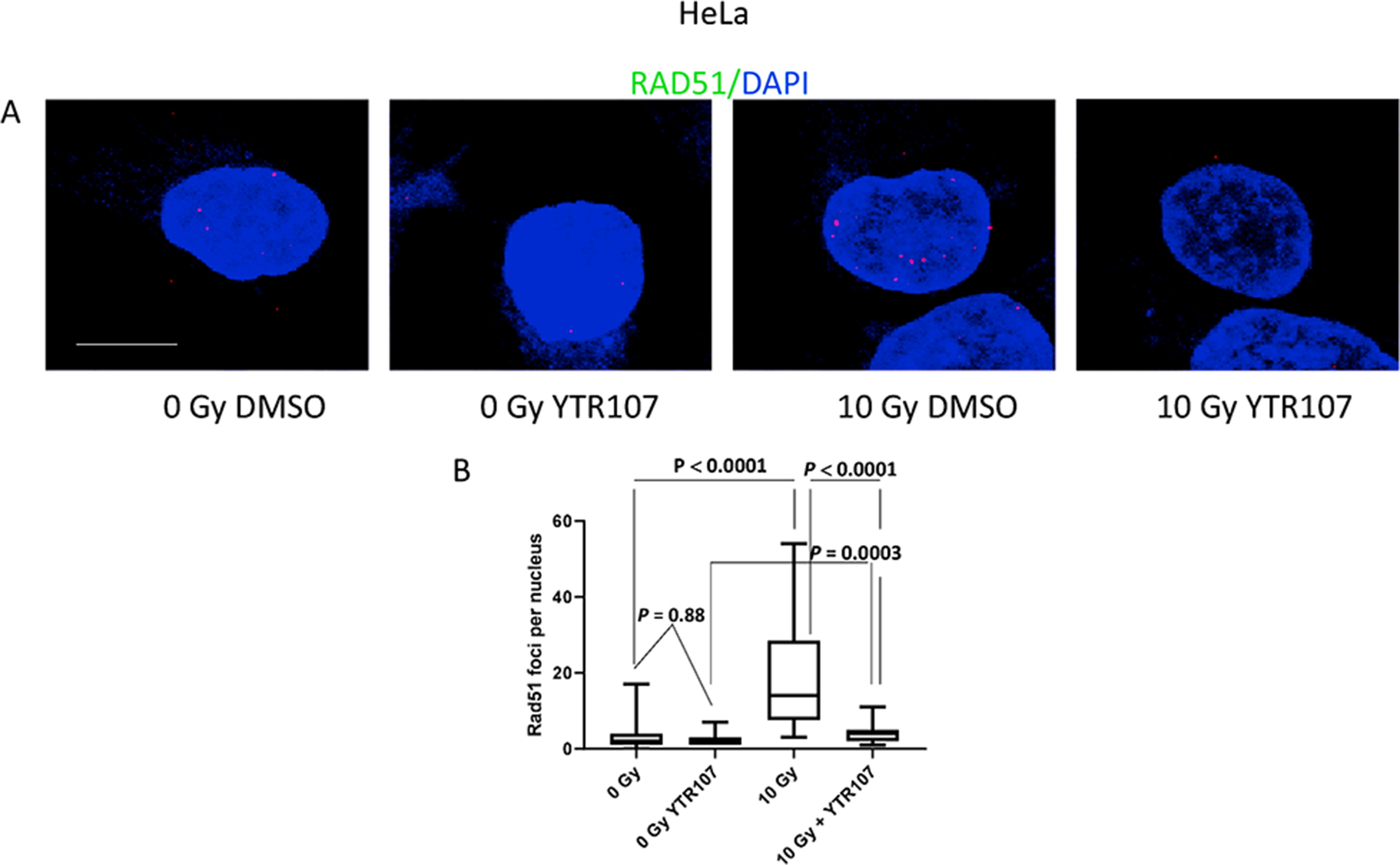

Supplement: Supp.Fig1 [file NIHMS1658200-supplement-Supp_Fig1.jpg]

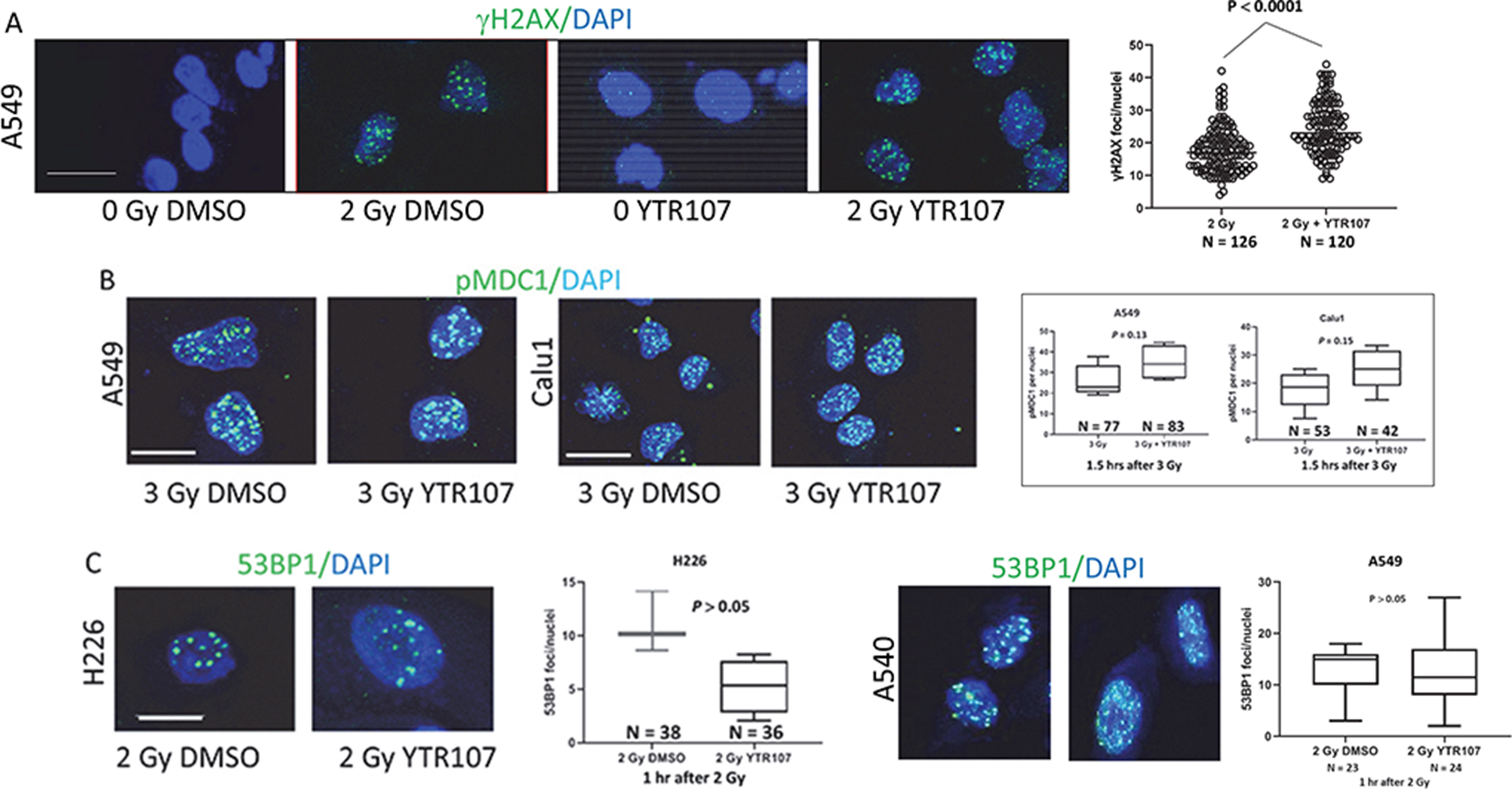

Supplement: Supp.Fig2 [file NIHMS1658200-supplement-Supp_Fig2.jpg]

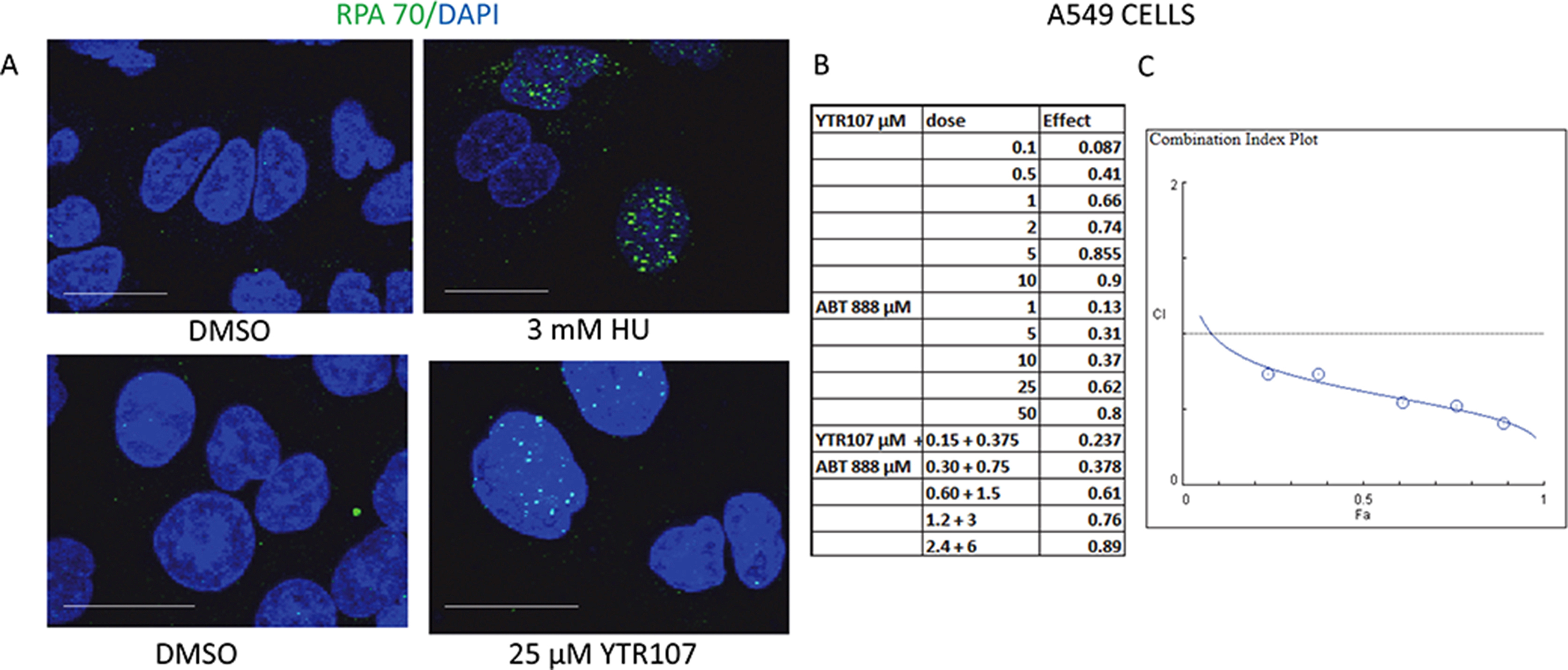

Supplement: Supp.Fig3 [file NIHMS1658200-supplement-Supp_Fig3.jpg]

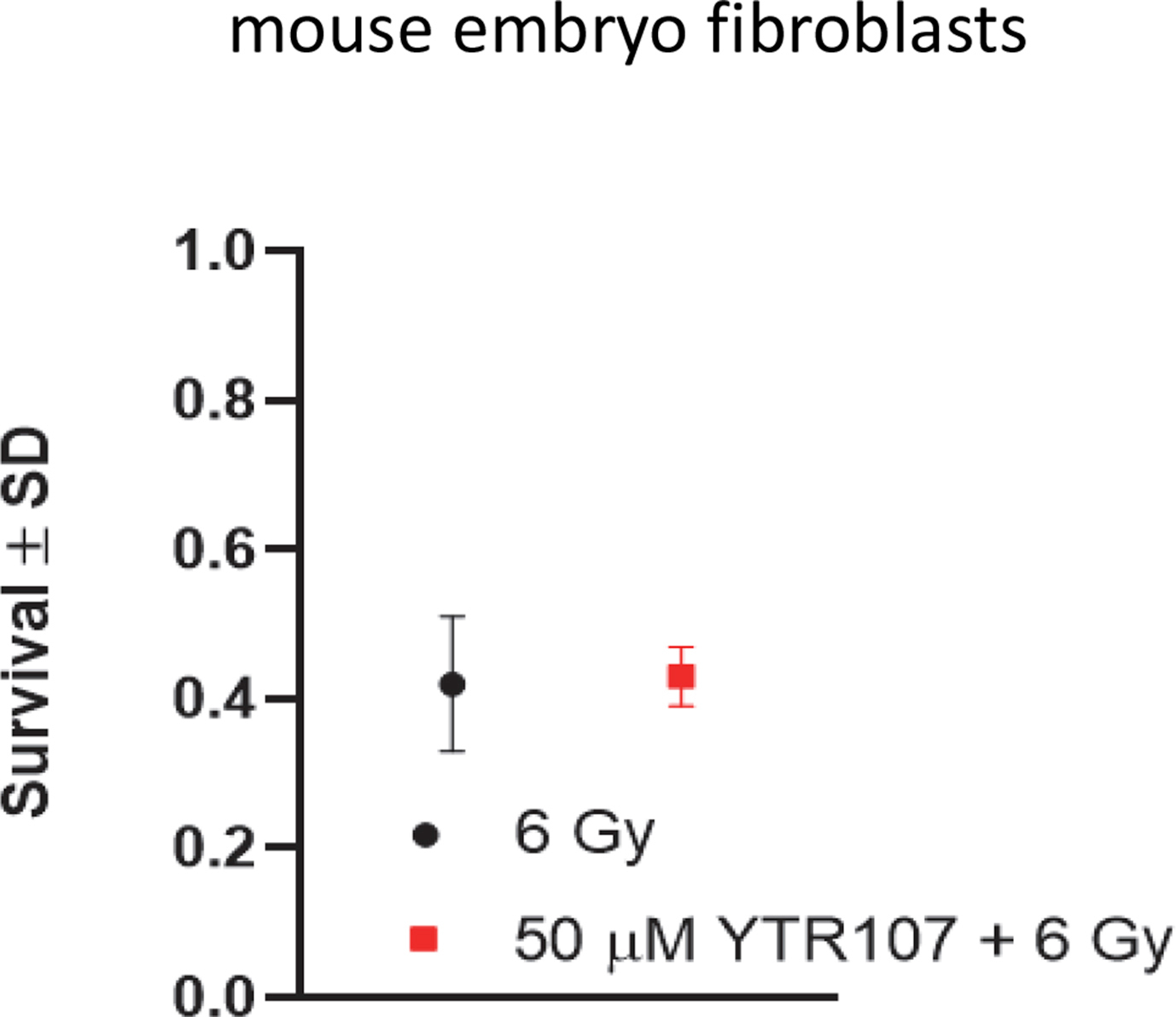

Supplement: Supp.Fi4 [file NIHMS1658200-supplement-Supp_Fi4.jpg]

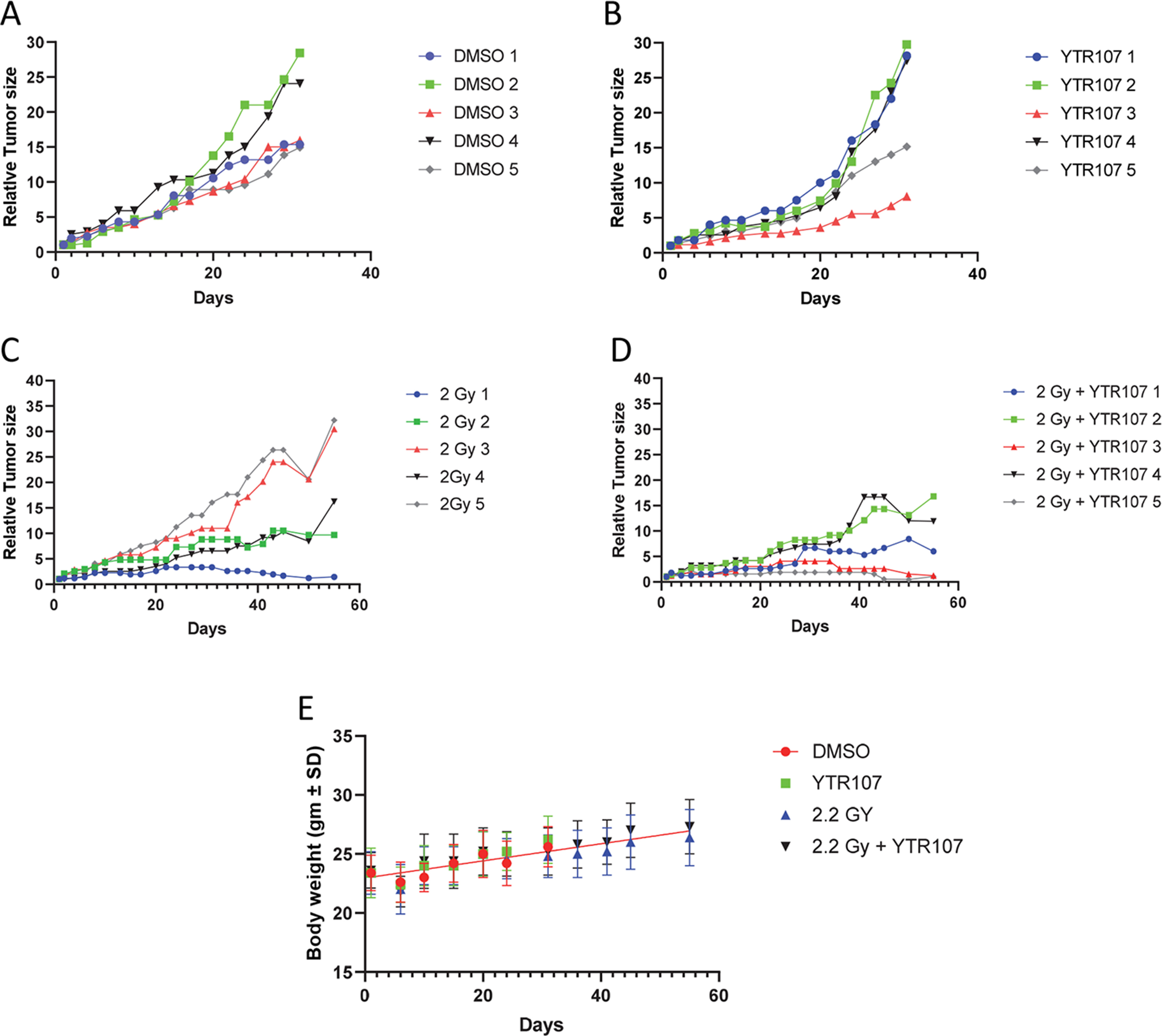

Supplement: Supp.Fig5 [file NIHMS1658200-supplement-Supp_Fig5.jpg]
